# *Bacillus clausii* for the Treatment of Acute Diarrhea in Children: A Systematic Review and Meta-Analysis of Randomized Controlled Trials

**DOI:** 10.3390/nu10081074

**Published:** 2018-08-12

**Authors:** Gianluca Ianiro, Gianenrico Rizzatti, Manuel Plomer, Loris Lopetuso, Franco Scaldaferri, Francesco Franceschi, Giovanni Cammarota, Antonio Gasbarrini

**Affiliations:** 1 Fondazione Policlinico Universitario A. Gemelli IRCCS-Università Cattolica del Sacro Cuore, 00143 Roma, Italy; gianenrico.rizzatti@gmail.com (G.R.); lopetusoloris@libero.it (L.L.); francoscaldaferri@gmail.com (F.S.); francesco.franceschi@unicatt.it (F.F.); giovanni.cammarota@unicatt.it (G.C.); antonio.gasbarrini@unicatt.it (A.G.); 2 Medical Affairs CHC Germany, Sanofi-Aventis Deutschland GmbH, Industriepark Höchst, D-65926 Frankfurt am Main, Germany; Manuel.Plomer@sanofi.com

**Keywords:** acute diarrhea, children, *Bacillus clausii*, efficacy, randomized controlled trials

## Abstract

Acute diarrhea is a burdensome disease with potentially harmful consequences, especially in childhood. Despite its large use in clinical practice, the efficacy of the probiotic *Bacillus clausii* in treating acute childhood diarrhea remains unclear. Our objective was to systematically review the efficacy of *Bacillus clausii* in the treatment of acute childhood diarrhea. The following electronic databases were systematically searched up to October 2017: MEDLINE (via PubMed/OVID), EMBASE (via OVID), Cochrane Central Database of Controlled Trials (via CENTRAL), Google Scholar, and ClinicalTrials.gov. Only randomized controlled trials were included. The overall effect for the meta-analysis was derived by using a random effects model. Six randomized controlled trials (1298 patients) met the eligibility criteria. Data arising from pooled analysis showed that *Bacillus clausii* significantly reduced the duration of diarrhea (mean difference = −9.12 h; 95% confidence interval [CI]: −16.49 to −1.75, *p* = 0.015), and the duration of hospitalization (mean difference = −0.85 days; 95% CI: −1.56 to −0.15, *p* = 0.017), compared with control. There was a trend of decreasing stool frequency after *Bacillus clausii* administration compared with the control group (mean difference = −0.19 diarrheal motions; 95% CI: −0.43 to −0.06, *p* = 0.14). *Bacillus clausii* may represent an effective therapeutic option in acute childhood diarrhea, with a good safety profile.

## 1. Introduction

Diarrhea refers to the abrupt onset of three or more loose or liquid stools per day [1]. More specifically, acute diarrhea is defined as an abnormally frequent discharge of semi-solid or fluid fecal matter from the bowel, lasting less than 14 days [2]. Although it is a preventable disease, acute diarrhea remains a major cause of morbidity and mortality in children worldwide, resulting in 525,000 deaths per year among those younger than five years. Most of these mortalities occur in developing countries [1]. Other direct consequences of diarrhea in children include growth faltering, malnutrition, and impaired cognitive development [3]. Acute diarrhea in children is caused by a wide range of pathogens—including viral, bacterial, and protozoal pathogens—which makes overcoming the high disease burden a large challenge [4].

Currently, the World Health Organization (WHO) recommends treatment of acute childhood diarrhea with oral rehydration salts (ORS) and continued feeding for the prevention and treatment of dehydration, as well as zinc supplementation to shorten the duration and severity of the diarrheal episode [1]. Probiotics are living micro-organisms that, upon ingestion in certain numbers, exert health benefits beyond inherent general nutrition [5]. It has been suggested that probiotics modulate the immune response, produce antimicrobial agents, and compete in nutrient uptake and adhesion sites with pathogens [6,7,8].

*Bacillus clausii* is a rod-shaped, non-pathogenic, spore-forming, aerobic, Gram-positive bacterium that is able to survive transit through the acidic environment of the stomach and colonize the intestine even in the presence of antibiotics [9]. Prospective clinical trials conducted in adult subjects found *Bacillus clausii* to be effective and safe in the treatment and prevention of acute diarrhea [10,11]. In a prospective, Phase II clinical trial of *Bacillus clausii* in 27 adult patients with acute diarrhea, the mean ± standard deviation (SD) duration of diarrhea decreased from 34.81 ± 4.69 min at baseline to 9.26 ± 3.05 (*p* < 0.0001) minutes per day after 10 days of *Bacillus clausii* therapy. The mean ± SD frequency of defecation also decreased from 6.96 ± 1.05 to 1.78 ± 0.50 (*p* < 0.0001) times per day, abdominal pain decreased from 3.22 ± 0.93 (severe) to 0.74 ± 0.71 (absent) (*p* < 0.0001), and stool consistency improved from 3.93 ± 0.38 (watery) to 1.22 ± 0.42 (soft) (*p* < 0.0001). No significant change in safety parameters was observed during treatment with *Bacillus clausii*. Thus, the study concluded that *Bacillus clausii* can potentially be effective in alleviating the symptoms of diarrhea without causing any adverse effects [11].

The European Society for Pediatric Gastroenterology, Hepatology, and Nutrition (ESPGHAN) and the European Society of Pediatric Infectious Diseases (ESPID) currently recommend the use of *Lactobacillus rhamnosus* GG and *Saccharomyces boulardii* in the management of children with acute diarrhea as an adjunct to rehydration therapy, whereas a recommendation for *Bacillus clausii* is missing due to limited data [12]. The aim of this paper is to systematically review randomized controlled trials that assessed the efficacy and safety of *Bacillus clausii* in the treatment of acute childhood diarrhea. According to our knowledge, no systematic reviews with meta-analyses addressing the effectiveness of *Bacillus clausii* in acute pediatric diarrhea have yet been published. We will focus only on studies using *Bacillus clausii* as a probiotic, because critics of using a meta-analytical approach to assess the efficacy of probiotics argue that beneficial effects of probiotics seem to be strain-specific.

## 2. Methods

### 2.1. Criteria for Considering Studies for this Review

We included randomized controlled trials conducted among children under 18 years of age with acute diarrhea (≤14 days). Patients in the experimental groups had to receive *Bacillus clausii* at any dose and in the following four bacterial stains: O/C, SIN, N/R, and T. Patients in the control groups had to receive either a placebo, an appropriate standard of care for acute diarrhea in lieu of the probiotic, or no treatmentcontrol. The designations of these bacterial strains are derived from their resistance to diverse antibiotics: O/C is resistant to chloramphenicol, SIN to neomycin and streptomycin, N/R to novobiocin and rifampin, and T to tetracycline [13].

The primary outcome measures were duration of diarrhea, stool frequency after intervention, and hospitalization duration. The secondary outcome measures were vomiting episodes, quality of life, and adverse events. All randomized controlled trials regardless of language or publication date or state (published, unpublished, in press, and in progress) were included in the review. Studies investigating probiotics other than *Bacillus clausii* (including synthetic microbiota suspensions), as well as those conducted in adult subjects or in children receiving *Bacillus clausii* for indications other than acute diarrhea were excluded. In vitro/vivo studies, observational studies, narrative/systematic reviews, case reports, letters, editorials, and commentaries were also excluded, but read to identify potential additional studies.

### 2.2. Search Strategy for Identification of Studies

The following electronic databases were systematically searched up to October 2017 for relevant studies: MEDLINE (via PubMed/OVID), EMBASE (via OVID), Cochrane Central Database of Controlled Trials (via CENTRAL), Google Scholar, and ClinicalTrials.gov (https://clinicaltrials.gov). The last literature search was conducted on 23 October 2017. The text word terms used were: *Bacillus clausii*; Enterogermina; probiotic; probiotics; diarrhea; diarrhoea; acute diarrhea; acute diarrhoea; diarrh *; children; child *; pediatric; and pediatr *. In addition, we hand-searched the bibliographies of papers of interest to provide additional references. Relevant meeting abstracts via EMBASE and the International Probiotic Conference were also hand-searched. When needed, we contacted the authors for additional data and clarification of study methods. Finally, the pharmaceutical company Sanofi-Aventis Group (Paris, France), which manufactures *Bacillus clausii* was contacted to identify further published and unpublished studies. No limit was imposed regarding the language of publication, and both studies published as full text or as abstracts at conferences/proceedings of scientific meetings were included in the review.

### 2.3. Study Selection

Titles and abstracts of publications identified according to the above described search strategy were independently screened by two reviewers (G.I. and G.R.). All potentially relevant articles were retained and the full text of these studies were examined to determine which studies satisfied the inclusion criteria. In the case of any differences of opinion or disagreements between the two reviewers, an adjudicator (A.G.) was consulted.

### 2.4. Data Extraction

Data extraction was carried out independently by two reviewers (G.I. and G.R.), using a data collection form designed for this review prepared in Microsoft Excel 2013 (Microsoft, Redmond, WA, USA). Discrepancies between the two reviewers were resolved by discussion. Information about the study design and outcomes was verified by all reviewers. Authors’ names, publication year, study design, study location, study duration, inclusion and exclusion criteria, interventions, type of comparator, number of patients, age and gender of included patients, outcomes, and adverse events were extracted from each study. To keep track of study references, EndNote version X7.71 (Thomson Reuters, New York, NY, USA) was used.

### 2.5. Quality Assessment

To assess the methodological quality of each study included in the review, two reviewers (G.I. and G.R.) independently performed a risk of bias assessment using the criteria (generation of allocation sequence; allocation concealment; blinding of investigators, participants, outcome assessors, and data analysts; intention-to-treat (ITT) analysis; and comprehensive follow-up) described by the Center for Reviews and Dissemination (CRD)’s guidance for undertaking reviews in health care (2009) [14]. For each criterion, the risk of bias was assessed answering the respective questions with ‘yes’, ‘no’, or ‘unclear’ and the overall quality of each study was rated « good », « fair » or «poor ».

### 2.6. Statistical Methods

Mean values and SDs of diarrhea duration, number of stools, and hospitalization duration were extracted to calculate the mean difference between the treatment and control groups for each of these outcomes. Overall effect for each meta-analysis was derived by using a random effects model, which takes between-study variation into account [15]. We also reported the corresponding 95% confidence intervals (CI) and *p*-values. Statistical heterogeneity between studies was assessed by using Cochran’s Q test and I-squared [16]. An I^2^ value of 0% indicates no observed heterogeneity, and larger values show increasing heterogeneity.

The risk of publication bias was assessed by visual inspection of Begg’s funnel plots. Formal statistical assessment of funnel plot asymmetry was also done using Egger’s regression asymmetry test and Begg’s adjusted rank correlation test [17]. All statistical analyses were conducted by using the metafor package (Maastricht University, Maastricht, NL, USA) [18]. *p*-Values < 0.05 were considered statistically significant.

## 3. Results

### 3.1. Characteristics of Included Studies

**Figure 1:**
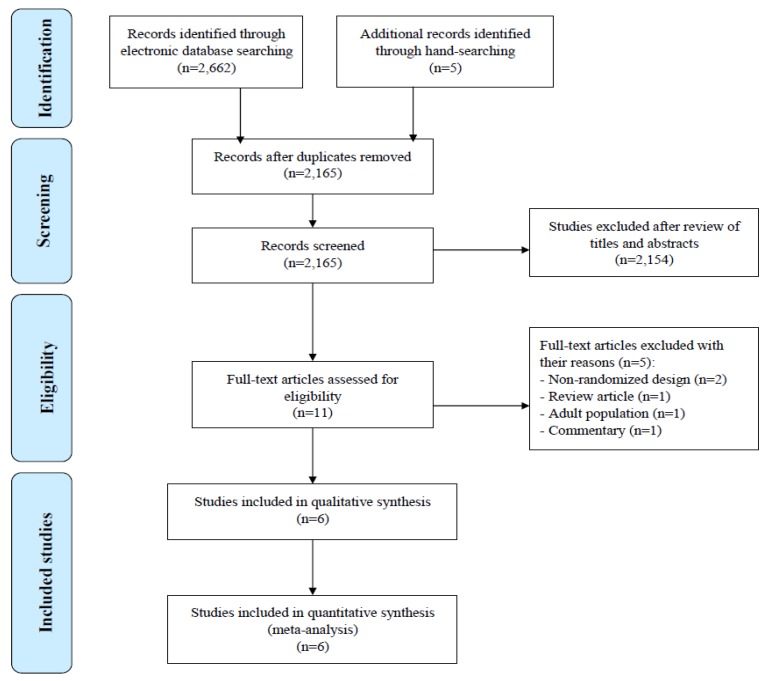
Flow diagram of the study selection process.

The literature search retrieved 2165 potential relevant citations. After carefully reviewing the titles and abstracts, 2154 citations were excluded. For the remaining 11 citations, full papers were obtained and reviewed. After a full-text assessment, six citations were included in the final database, and five excluded for the following reasons: two studies were non-randomized, one study was conducted in an adult population, one was a review article, and one was a commentary. The flow diagram of the study selection process is given in Figure 1.

Table 1 summarizes the characteristics of the six randomized controlled trials included in the review, which were published between 2007 and 2015. Of these, one was performed in Italy [19], one in Kenya [20], one in the Philippines [21], and three in India [22,23,24]. Three of the included studies were published as original articles [19,23,24], one as a meeting abstract [21], one as a Master’s dissertation [20], and one as a clinical study report [22]. Of the six studies, two were conducted in a multicentric setting [19,22]. All six studies included an outcome for diarrhea duration, four included an outcome for stool frequency [19,20,22,24], and three included an outcome for duration of hospitalization [20,21,23].

Overall, 1298 patients were enrolled in the six selected studies. Among these, 467 patients were treated with *Bacillus clausii*. In the Canani et al. (2007) study [19], patients were allocated to six different groups: a control group (*n* = 92), a group treated with *Bacillus clausii* (*n* = 100), a group treated with *Lactobacillus casei* (*n* = 100), a group treated with *Saccharomyces boulardii* (*n*=91), a group treated with *Lactobacillus delbrueckii var bulgaricus*, *Lactobacillus acidophilus*, *Streptococcus thermophilus*, *Bifidobacterium bifidum* (*n* = 97), and a group treated with *Enterococcus faecium* (*n* = 91). All groups, with the exception of the control group and the group receiving *Bacillus clausii* were excluded from this meta-analysis. Thus, in total, 919 patients were included in the meta-analysis (467 in the experimental group and 452 in the control group). The age of the patients ranged from 3 months to 12 years. Four studies enrolled inpatients [20,21,23,24], whereas two enrolled outpatients [19,22].

In all six clinical trials, the control group was treated with ORS. In the Canani et al. (2007) study [19], the control group (*n* = 92) was given an oral rehydration solution for 3 to 6 h and then fed with a full-strength milk formula containing lactose or cows’ milk, depending on age. In the three Indian studies, the control group (*n* = 132 in the Lahiri trial [22]; *n* = 80 in the Lahiri, D’Souza et al. trial [24]; and *n* = 62 in the Lahiri, Jadhav et al. trial [23]) received ORS with zinc supplementation. The control group in the Urtula and Dacula (2008) study (*n* = 35) received ORS alone [21]. Finally, the control group in the Maugo (2012) study (*n* = 51) received in addition to zinc sulfate and ORS, one vial twice daily of a placebo packaged in identical looking vials containing sterile water [20]. Concerning the interventions in the experimental group, in one study, the daily dosage of *Bacillus clausii* was 1 × 10^9^ colony-forming units (CFU) administrated twice daily [19], while in four other studies, children were administered 2 × 10^9^ CFU of *Bacillus clausii* twice daily [20,22,23,24], and in the Urtula and Dacula (2008) trial, 2 × 10^9^ or 4 × 10^9^ CFU of *Bacillus clausii* were administrated per day, depending on the age of the children [21]. In all studies, the experimental group received ORS in addition to *Bacillus clausii* therapy. Moreover, zinc supplementation was also added to the treatment of the experimental group in four studies [20,22,23,24]. The duration of the interventions was five days in all clinical trials, with the exception of the Urtula and Dacula (2008) trial [21] which treated patients for three days.

### 3.2. Risk of Bias within Included Studies

The methodological quality of the clinical trials varied (Table 2). Three studies [19,20,21] were rated as adequate for both generation of the allocation sequence and allocation concealment. In the remaining three studies, the method used for allocation sequence and allocation concealment was unclear [22,23,24]. In only one study [20], care providers, participants, and outcome assessors were blind to treatment allocation. In the Canani et al. (2007) study [19] and in the Lahiri (2008) trial [22], analyses were conducted on an ITT basis. Three studies [21,23,24] were unclear for an ITT analysis, and the Maugo (2012) trial [20] did not include an ITT analysis. Loss to follow-up was adequate in two studies [20,22], and was unclear in the remaining four studies [19,21,23,24]. The overall quality was assessed, with two studies [19,20] rated as ‘good’ (low risk for bias), two other studies [21,22] which were susceptible to some bias rated as ‘fair’, and the remaining two studies [23,24] were rated as ‘poor’ (high risk for bias).

### 3.3. Primary Findings

**Figure 2:**
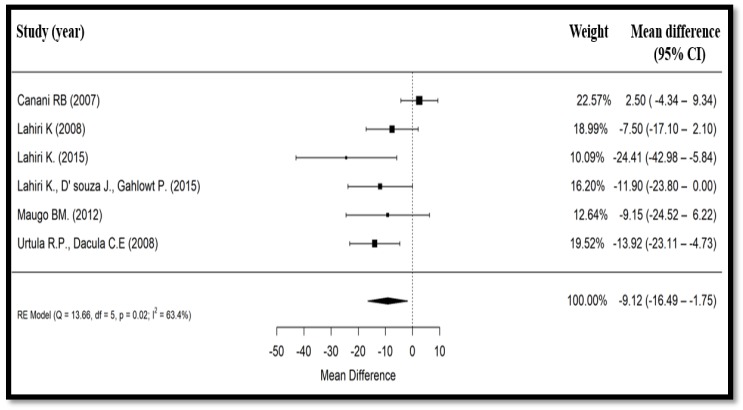
Forest plot showing effect of *Bacillus clausii* on mean duration of diarrhea. CI, confidence interval, RE, random effects.

All six studies contained data on the duration of diarrhea. Compared to the control group (*n* = 441), the change in diarrhea duration in patients treated with *Bacillus clausii* (*n* = 457) ranged from −24.4 to +2.5 h among included studies. In the Canani et al. (2007) trial [19], duration of diarrhea was expressed as median (interquartile range [IQR]) duration, whereas in three studies [20,21,22], it was expressed as mean (SD) duration, and in two studies [23,24], it was simply expressed as mean duration. According to the Cochrane Reviewers’ Handbook 4.2.2 (2004) [25] and assuming normal distribution, median duration of diarrhea in the Canani et al. (2007) study [19] was treated as a mean value, and the width of IQR was considered as 1.35 × SD. After this conversion, a meta-analysis of the six randomized controlled trials (898 participants) showed a significant reduction in the duration of the diarrhea (mean difference = −9.12 h, 95% CI: −16.49 to −1.75) for those treated with *Bacillus clausii* compared to ORS with or without zinc supplementation (*p* = 0.015) (Figure 2). The heterogeneity test for diarrhea duration showed a substantial heterogeneity between the six studies (Cochrane’s Q test, *p* = 0.02, *I*^2^ = 63.4%).

**Figure 3:**
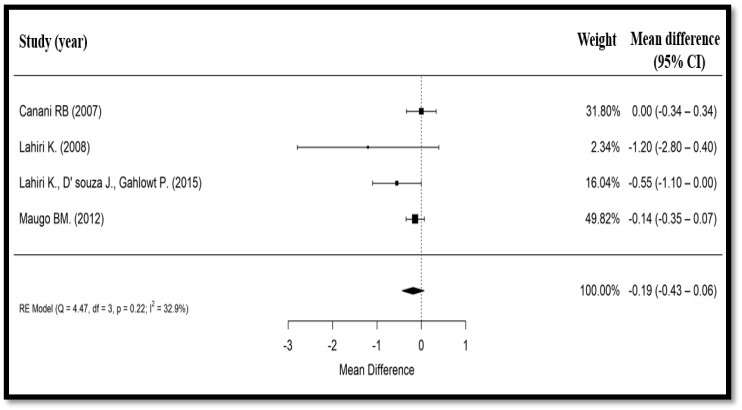
Forest plot showing effect of *Bacillus clausii* on mean stool frequency. CI, confidence interval, RE, random effects.

Four studies (697 participants) evaluated stool frequency after intervention [19,20,22,24]. In the Canani et al. (2007) trial [19], daily stool frequency was expressed as median (IQR), and it was evaluated from the first day of *Bacillus clausii* administration up to day 7. In the Maugo (2012) study [20], daily diarrheal output was expressed as mean (SD), and it was also evaluated from day 1 of *Bacillus clausii* administration up to day 7. In the Lahiri (2008) trial [22], daily diarrheal output was expressed as both mean (SD) and median (range) values, and it was evaluated from day 1 of *Bacillus clausii* administration up to day 6. Finally, in the Lahiri, D’Souza et al. (2015) study [24], stool frequency was expressed as a mean value, and it was assessed before and after treatment with *Bacillus clausii*. Similarly to the duration of diarrhea, median stool frequency in the Canani et al. (2007) study [19] was treated as a mean value, and the width of IQR was considered as 1.35 × SD [25]. Pooling the results of the four trials showed that *Bacillus clausii* reduces the stool frequency after intervention (mean difference = −0.19 diarrheal motions, 95% CI: −0.43 to −0.06, *p* = 0.14) compared with the control group which received ORS with or without zinc supplementation (Figure 3). The heterogeneity test for stool frequency after intervention revealed a slight heterogeneity between the four trials (Cochrane’s Q test, *p* = 0.22, *I*^2^ = 32.9%).

**Figure 4:**
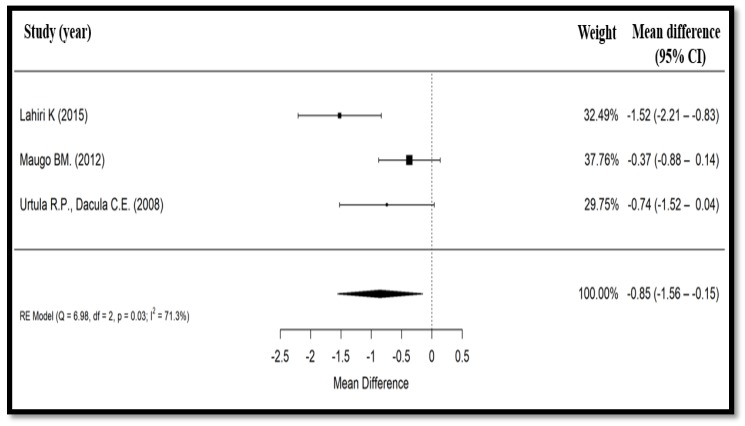
Forest plot showing effect of *Bacillus clausii* on mean duration of hospitalization. CI, confidence interval, RE, random effects.

Finally, duration of hospitalization was assessed in three studies [20,21,23] among 291 patients. In the Maugo (2012) study [20], hospitalization duration was expressed as mean (SD), whereas in the two other trials [21,23], it was simply expressed as mean. Based on the results of these three clinical trials [20,21,23], there was a significant reduction in the duration of hospitalization (mean difference = −0.85 days, 95% CI: −1.56 to −0.15) for those treated with *Bacillus clausii* compared to ORS with or without zinc (*p* = 0.017) (Figure 4). The heterogeneity test for duration of hospital stay showed a substantial heterogeneity between the three studies (Cochrane’s *Q* test, *p* = 0.03, *I*^2^ = 71.3%).

### 3.4. Secondary Findings

Two clinical trials [19,22] included an outcome related to the incidence and/or duration of vomiting episodes among 447 patients. In the Canani et al. (2007) trial [19], both median (IQR) duration of vomiting and the number (%) of children experiencing vomiting episodes were similar in the group treated with *Bacillus clausii* (*n* = 100) and in the control group (*n* = 92). In the control group, 34 children (37%) experienced vomiting episodes versus 32 children (32%) in the *Bacillus clausii* group (*p* = 0.47). Similarly, the median (IQR) vomiting duration was 2 (1–2) days in the control group versus 1.5 (1–2) days in the group treated with *Bacillus clausii* (*p* = 0.25). In the Lahiri (2008) study [22], the mean ± SD number of vomiting episodes on day 4 of treatment was 0.1 ± 0.6 in the *Bacillus clausii* + ORS group (*n* = 129) versus 0.2 ± 0.6 in the ORS group (*n* = 126). Hence, the difference in the mean number of vomiting episodes was not statistically significant between the two groups (*p* = 0.79).

The studies [19,22] did not report any serious adverse effects related to *Bacillus clausii*. According to Canani and colleagues [19], treatment by *Bacillus clausii* was well tolerated, and no adverse events were observed. In the Lahiri (2008) trial [22], 40/129 patients (31%) from the *Bacillus clausii* + ORS group and 39/126 patients (31%) from the ORS group experienced undesirable side effects. There was no statistically significant difference in the number of patients experiencing adverse events between the two groups (*p* = 0.48). Vomiting was the most reported adverse event in both the *Bacillus clausii* + ORS group (20/129; 15.5%) and the ORS group (17/126; 13.5%).

Outcomes related to quality of life were not reported in any of the studies included in the meta-analysis.

### 3.5. Publication Bias

The publication bias was assessed by using a funnel plot depicting the mean differences in duration of diarrhea, stool frequency, and duration of hospital stay against their effect sizes as a measure of precision. A slight asymmetry was seen in Begg’s funnel plot for duration of diarrhea, resulting in evidence of publication bias (Egger’s test, *p* = 0.02). In contrast, duration of hospital stay and stool frequency showed neither asymmetry nor evidence for publication bias (Egger’s test, *p* = 0.55 for hospitalization duration and *p* = 0.11 for stool frequency).

## 4. Discussion

We conducted a systematic review and a meta-analysis of randomized controlled trials to estimate the efficacy of *Bacillus clausii* in the treatment of acute diarrhea in children. Results of this systematic review indicate that *Bacillus clausii* combined with ORS might significantly reduce the duration of acute childhood diarrhea and the duration of hospital stay compared to ORS alone.

To our knowledge, this is the first systematic review focusing on randomized controlled trials of *Bacillus clausii* in acute childhood diarrhea. In this review, the duration of diarrhea was reduced by a mean of 9.12 h with *Bacillus clausii* treatment compared to controls (*p* = 0.015). These findings were replicated in a prospective, phase II, Indian clinical study conducted among 27 adult patients with acute diarrhea treated with 2×10^9^ CFU of *Bacillus clausii* twice daily for a duration of 10 days, in which mean ± SD duration of diarrhea decreased from 34.81 ± 4.69 min at baseline to 9.26 ± 3.05 (*p* < 0.0001) minutes per day after 10 days of *Bacillus clausii* administration [11]. In contrast, in the Canani et al. (2007) trial [19], it was found that the duration of diarrhea in patients receiving *Bacillus clausii* was similar to that in the group receiving only oral rehydration, with an estimated difference of one hour between the control group and the group treated with *Bacillus clausii* (*p* = 0.76). The difference between the overall results of our meta-analysis and the results of the Canani et al. (2007) trial [19] may be due to the difference in the prescribed dosage of *Bacillus clausii* in the different randomized controlled trials and the zinc supplementation provided in some study protocols [20,22,23,24]. In the other studies, children were administered 4 × 10^9^ CFU of *Bacillus clausii* per day [20,22,23,24], while in the Canani et al. (2007) trial [19], children received 2 × 10^9^ CFU of *Bacillus clausii* per day, which also corresponds to the prescribed dosage of *Bacillus clausii* in the younger children of the Urtula and Dacula (2008) study [21].

Our results also showed that administration of *Bacillus clausii* preparations significantly reduced the duration of hospitalization by a mean of 0.85 days compared to controls (*p* = 0.017). The reduction of hospital stay by *Bacillus clausii* is important considering that in low-income countries, children under three years old experience on average three episodes of diarrhea every year [1]. Moreover, a 2008 study set in in Vellore, India, in 439 children under the age of five years found that median household expenditures incurred per diarrheal episode ranged from 2.2% to 5.8% of the household’s annual income [26]. Similarly, a 2013 cross-sectional study set in Bolivia and conducted among 1107 caregivers of pediatric patients (<5 years of age) with diarrhea found that 45% of patients’ families paid ≥1% of their annual household income for a single diarrheal episode [27]. Thus, diarrheal disease in children constitutes a considerable worldwide economic burden. The results of this systematic review are of particular importance, since these reductions in the length of hospital stay and duration of diarrhea that were obtained with *Bacillus clausii* in our analysis may offer significant social and economic benefit in the treatment of acute childhood diarrhea, particularly in low- and middle-income countries. In addition, in the Lahiri, Jadhav et al. (2015) study [23], treatment with *Bacillus clausii* reduced total treatment costs by 472 Indian rupees compared to ORS alone. Further studies may be needed to clarify the cost-effectiveness of *Bacillus clausii* preparations in treating children with acute diarrhea.

The effect of *Bacillus clausii* on stool frequency reduction compared to ORS alone did not reach statistical significance after pooling the results of four clinical trials (*p* = 0.14). This result could have different explanations. First, assessing such a specific outcome, as stool frequency can be challenging. Moreover, these four studies [19,20,22,24] differed in sample size, study design, and treatment protocols. Consequently, large studies might be needed to clarify the efficacy of *Bacillus clausii* on stool frequency reduction in acute pediatric diarrhea.

Our systematic review suggested that treatment with *Bacillus clausii* is well tolerated, without causing serious adverse events. This finding is consistent with the safety results of the prospective, Phase II clinical trial conducted in 27 adult patients with acute diarrhea which found no significant change in safety parameters during treatment with *Bacillus clausii* [11]. Additionally, in a 2004 single-center, double-blind, prospective, randomized, placebo-controlled study performed in 120 consecutive *Helicobacter pylori*-positive adult patients free from gastrointestinal symptoms, it was found that *Bacillus clausii* treatment during and after a standard seven-day anti-*Helicobacter pylori* regimen was also associated with lower incidence of self-reported side-effects and a better tolerability to multiple antibiotic treatment when compared with placebo (*p* < 0.05) [10].

Between-trial heterogeneity was detected for diarrhea duration and duration of hospital stay. This heterogeneity among the included studies could be partially explained by trials at high/unclear risk of bias for sequence generation, allocation concealment, and/or blinding. Indeed, only one included study was double-blinded [20], whereas the five other studies were either single-blinded [19], open-label [22,23,24], or had unclear blinding [21]. However, a slight heterogeneity for stool frequency after intervention was detected, reflecting an apparent effect of *Bacillus clausii* administration on stool frequency reduction compared with the control group.

Several mechanisms have been proposed to explain the effect of *Bacillus clausii* against acute childhood diarrhea. Urdaci and colleagues found *Bacillus clausii* to possess antimicrobial and immunomodulatory activities. Moreover, *Bacillus clausii* strains were found to release antimicrobial substances in the medium, and this was observed during stationary growth phase and coincided with sporulation. These substances were active against Gram-positive bacteria, in particular against *Staphylococcus aureus*, *Enterococcus faecium*, and *Clostridium difficile*. The antimicrobial activity of *Bacillus clausii* was resistant to subtilisin, proteinase K, and chymotrypsin treatment, whereas it was sensitive to pronase treatment [28]. The ability of *Bacillus clausii* spores to germinate during gastrointestinal transit and grow as vegetative cells both in the presence of bile and under limited oxygen availability was also described in an experimental study by Cenci et al. (2006) [29]. Additionally, *Bacillus clausii* O/C supernatant was found to reduce the cytotoxic effects of *Clostridium difficile* and *Bacillus cereus* toxins through the secreted alkaline serine M-protease [30]. Finally, the production of vitamin B2 by *Bacillus clausii* (strains O/C, N/R, SIN, and T) was compared with that of other probiotics in an in vitro agar-diffusion assay, and it was found that only *Bacillus clausii* and *Bacillus subtilis* permitted the growth of MS0057, a riboflavin-auxotrophic mutant of *Bacillus cereus*, which indicates secretion and diffusion of vitamin B2 in the solid medium [31]. These results are consistent with the beneficial effects evidenced for *Bacillus clausii* preparations in our study.

Our review had limitations that must be considered while interpreting our results. Three studies had unclear sequence generation and allocation concealment, five had inadequate or unclear blinding, and four were unclear for or had no ITT analysis. In addition, the definition of diarrhea, the termination of diarrhea, and inclusion and exclusion criteria varied among the included studies. In our meta-analysis, we also noticed publication bias detected for diarrhea duration. A key strength of the study comes from the fact that only a clearly defined probiotic micro-organism mix of four *Bacillus clausii* strains was assessed. Moreover, all treatments received by the control groups in the included studies were standardized consisting of ORS with or without zinc supplementation. Only the control group in the Maugo (2012) study received a placebo [20].

In summary, our results indicate that *Bacillus clausii* might represent an effective therapeutic option in acute childhood diarrhea, with a good safety profile. One limitation of this meta-nalysis is represented by the heterogeneity we found among studies, that prevent us from drawing definitive conclusions. Further, well designed studies are needed to confirm our findings.

## Figures and Tables

**Table 1:** Characteristics and results of included studies.

Authors, Publication Year (Country)	Study Design	Number of Treated Patients (I/C)	M/F (In %)	Age	Intervention vs. Comparator (Dosage and Duration)	Outcome Measures	Follow-Up	Main Results
Canani et al., 2007 (Italy) [19]	Prospective, multicenter, single-blind, randomized, controlled	100/92	47/53	Median: 18 months	1 × 10^9^ CFU of *Bacillus clausii* bid for 5 days + ORS for 3 to 6 h vs. ORS for 3 to 6 h (followed by full strength formula of lactose or cows’ milk, depending on age, in both groups)	Total duration of diarrhea, number of stools/day and their consistency, incidence and median duration of vomiting, fever (>37.5 °C), number of hospital admissions, safety and tolerability	Day 1 to day 7	Median duration of diarrhea in patients receiving *Bacillus clausii* (118 h) similar to control group (115 h), with an estimated difference of 1 h between both groups (*p* = 0.76). All other outcomes were also similar in both groups. *Bacillus clausii* was well tolerated, with no observed adverse events.
Lahiri, 2008 (India) [22]	Phase III, controlled, open-label, randomized, parallel-group, multicenter, comparative	132/132	54.5/45.5	Mean (SD): 1.6 (1.0) years	2 × 10^9^ CFU of *Bacillus clausii* bid + ORS + 20 mg/day of zinc supplement, for 5 days vs. ORS + 20 mg/day of zinc supplement, for 5 days	Duration of diarrhea, mean number of daily stools, effect on consistency of stools, vomiting episodes per day, reported adverse events, parents’ overall global assessment of tolerability at end of treatment period	Day 6 to day 10 (after end of study treatment)	Mean (SD) duration of diarrhea lower in the experimental group (48.6 (38.2) h), vs. control group (56.1 (40) h; *p* = 0.13). Difference in the mean (SD) number of stools until recovery statistically not significant (*p* = 0.19); trend favoring the experimental group (7.4 (6.5) motions vs. 8.6 (6.5) motions in control group).
Lahiri, Jadhav et al., 2015 (India) [23]	Open-label, prospective, randomized, controlled	69/62	63.4/36.6	6 months to 12 years	2 × 10^9^ CFU of *Bacillus clausii* bid + ORS + zinc, for 5 days vs. ORS + zinc for 5 days	Mean duration of diarrhea, mean duration of hospitalization, frequency of diarrhea, direct and indirect costs	At 6, 12, 24, 36, 48, 60, and 72 h	Mean duration of diarrhea 22.64 h and mean duration of hospital stay 2.78 days in the *Bacillus clausii* group vs. 47.05 h and 4.30 days, respectively, in the control group (*p* < 0.01 for diarrhea duration). Treatment with *Bacillus clausii* reduced total treatment costs by 472 Indian rupees compared to ORS alone.
Lahiri, D’Souza et al., 2015 (India) [24]	Open-label, prospective, randomized, controlled	80/80	52.5/47.5	Up to 6 years	2 × 10^9^ CFU of *Bacillus clausii* bid + ORS + zinc, for 5 days vs. ORS + zinc for 5 days	Mean duration of diarrhea, mean stool frequency, % of children with no dehydration, % of children benefiting from breastfeeding	At 6, 12, 24, 36, 48, 60, and 72 h	Mean (SD) duration of diarrhea 22.26 h and mean stool frequency 1.15 in the *Bacillus clausii* group vs. 34.16 h and 1.70, respectively in control group (*p* < 0.05).
Maugo, 2012 (Kenya) [20]	Randomized, double-blind, placebo- controlled	51/51	51.1/48.9	Mean (SD): *Bacillus clausii* group: 11.3 (5.3) and control group: 11.9 (6.4) months	2 × 10^9^ CFU of *Bacillus clausii* bid + ORS + zinc sulfate, for 5 days vs. zinc sulfate + ORS + 1 vial bid of a placebo packaged in identical looking vials containing sterile water, for 5 days	Mean duration of diarrhea, mean duration of hospitalization, mean reduction of the number of diarrheal episodes per day	Day 1 to day 7	Mean (SD) duration of diarrhea in *Bacillus clausii* group was shorter (77.59 (34.10) h) than placebo group (86.74 (40.16) h), with mean absolute difference between groups of 9.15 h (*p* = 0.248). Significant decrease in mean number of diarrheal motions on day 3 (2.74 (1.81) motions in the *Bacillus clausii* group vs. 3.80 (2.70) motions in placebo group, mean absolute difference = 1.05 motions; *p* = 0.033) and day 4 (1.45 (1.13) motions in the *Bacillus clausii* group vs. 2.35 (2.19) motions in placebo group, mean absolute difference = 0.9 motions; *p* = 0.018) in the *Bacillus clausii* group vs. placebo group.
Urtula and Dacula, 2008 (The Philippines) [21]	Monocentric, randomized, controlled	35/35	NR	NR	2 × 10^9^ or 4×10^9^ CFU of *Bacillus clausii* per day, depending on the age of the children + ORS, for 3 days vs. ORS for 3 days	Mean duration of diarrhea, mean duration of hospitalization, mean frequency of stools	After day 3 of therapy, and upon discharge	Mean (SD) duration of diarrhea significantly shorter in the *Bacillus clausii* group (69.84 (16.84) h) than in control group (83.76 (22.05) h) (*p* = 0.005), with absolute difference of duration of diarrhea between groups of 13.92 h. Mean duration of hospital stay was also shorter favoring *Bacillus clausii* group (59.0 h vs. 76.8 h) (*p* = 0.063).

bid, twice daily; C, control; CFU, colony-forming units; F, female; h, hour; I, intervention; M, male; NR, not reported; ORS, oral rehydration salts; SD, standard deviation; vs., versus.

**Table 2:** Risk of bias assessment.

Authors and Publication Year	Was Randomization Carried Out Appropriately?	Was the Concealment of Treatment Allocation Adequate?	Were the Groups Similar at the Outset of the Study in Terms of Prognostic Factors?	Were the Care Providers, Participants and Outcome Assessors Blind to Treatment Allocation?	Were There any Unexpected Imbalances in Drop-Outs between Groups?	Is There any Evidence to Suggest that the Authors Measured More Outcomes than They Reported?	Did the Analysis Include an Intention-To-Treat Analysis? If So, Was This Appropriate and Were Appropriate Methods Used to Account for Missing Data?	Overall Study Quality
Canani et al., 2007 [19]	Yes	Yes	Yes	No	No	No	Yes/Yes	Good
Lahiri, 2008 [22]	Unclear	Unclear	Unclear	No	No	Unclear	Yes/Yes	Fair
Lahiri, Jadhav et al., 2015 [23]	Unclear	Unclear	Unclear	No	Unclear	No	Unclear	Poor *
Lahiri, D’Souza et al., 2015 [24]	Unclear	Unclear	Unclear	No	Unclear	No	Unclear	Poor *
Maugo, 2012 [20]	Yes	Yes	Yes	Yes	No	No	No	Good
Urtula and Dacula, 2008 [21]	Yes	Yes	Yes	Unclear	Unclear	No	Unclear	Fair

* Risk of bias was classified according to the Centre for Reviews and Dissemination (CRD) [14], based on the information available in the publications. However, the principle investigator was contacted directly and confirmed the validity of the data quality, providing the authors with confidence that the risk for bias can be considered as ‘fair’.
